# The Roles of β-Integrin of Chinese Shrimp (*Fenneropenaeus chinensis*) in WSSV Infection

**DOI:** 10.3390/ijms18071465

**Published:** 2017-07-07

**Authors:** Xiaoqian Tang, Fude Zhai, Xiuzhen Sheng, Jing Xing, Wenbin Zhan

**Affiliations:** 1 Laboratory of Pathology and Immunology of Aquatic Animals, Ocean University of China, 5 Yushan Road, Qingdao 266003, China; tangxq@ouc.edu.cn (X.T.); aquamedic@ouc.edu.cn (F.Z.); xzsheng@ouc.edu.cn (X.S.); xingjing@ouc.edu.cn (J.X.); 2 Laboratory for Marine Fisheries Science and Food Production Processes, Qingdao National Laboratory for Marine Science and Technology, No. 1 Wenhai Road, Aoshanwei Town, Jimo, Qingdao 266071, China

**Keywords:** β-integrin, *Fenneropenaeus chinensis*, white spot syndrome virus, blocking assay, gene silence

## Abstract

Our previous study demonstrated that an integrin β subunit of Chinese shrimp (*Fenneropenaeus chinensis*) (FcβInt) plays an important role in white spot syndrome virus (WSSV) infection. In the present work, in order to further elucidate the potential role of FcβInt in WSSV infection, the recombinant extracellular domain of β integringene of *F. Chinensis* (rFcβInt-ER) was expressed in *Escherichia coli* BL21 (DE3), and the eukaryotic expression plasmid PcDNA3.1-FcβInt-ER (PFcβInt-ER) was also constructed. Far-western blotting was performed to determine the binding specificity of rFcβInt-ER to WSSV envelope proteins, and results showed that rFcβInt-ER was able to specifically interact with rVP31, rVP37, rVP110 and rVP187. Moreover, the blocking effects of mouse anti-rFcβint-ER antibodies were both detected in vivo and in vitro. The ELISA and Dot-blotting in vitro assays both showed that mouse anti-rFcβInt-ER antibodies could partially block the binding of WSSV to the hemocyte membrane of *F. chinensis*. In the in vivo assays, the mortality of shrimp injected with WSSV mixed with anti-rFcβInt-ER antibodies was delayed, and was lower than in the control group. While the shrimp were intramuscularly injected with PFcβInt-ER, transcripts of PFcβInt-ER could be detected in different shrimp tissues within 7 days, and the mortality of shrimp injected with PFcβInt-ER was also delayed and lower compared with the control group post WSSV challenge. Furthermore, gene silencing technology was also used to verify the effect of FcβInt in WSSV infection, and results showed that the expression levels of the WSSV immediate early gene *iel*, early gene *wsv477*, and late gene *VP28* and the mortality of *F. Chinensis* were all significantly decreased in the FcβInt knock-down hemocyctes compared to the control group. Taken together, these results suggest that FcβInt plays important roles in WSSV infection.

## 1. Introduction

Integrins are a superfamily of cell adhesion receptors that consist of totally distinct α and β subunits, with each subunit composed of an extracellular domain, a transmembrane spanning region and a small cytoplasmic domain [1]. Integrins are widely expressed in all metazoans and can mediate cell to cell, cell to extracellular matrix, and cell to pathogen interactions [2]. Multiple ligands could be recognized by integrins, and the interaction of integrins with their ligands induce a large variety of signal transduction to modulate cell behaviors [3]. In mammals, the function of integrins in regulation of cell adhesion, migration, proliferation, and apoptosis have been extensively studied [4]. Moreover, as important cell surface receptors, integrins play an important role in the process of infection for a large number of viruses, such as adenovirus, hantavirus, and herpesvirus [5,6,7,8]. However, in the case of invertebrates, knowledge of the functions of integrins in virus infection and the interactions between pathogen and integrins is still limited.

As an important and indispensable receptor on the cell membrane, the integrin can also bind to proteins that contain canonical integrin-binding motifs, such as Arginine-glycine-aspartic acid (RGD), leucine-aspartate-valine (LDV) and Tyrosine-glycine-leucine (YGL) [9]. RGD has been identified as a general integrin-binding motif, and can be recognized by over 20 known integrins. Several viruses and bacteria that contain canonical integrin-binding motifs in their surface could take advantage of this family of proteins to gain access to the cell [10]. It has been proven that many white spot syndrome virus (WSSV) envelope proteins possess RGD sequences, such as VP31, VP36A, VP36B, VP90, VP110, VP136, VP187 and VP281 [9,11]. Recent studies have also shown that integrin β-subunits (β-integrin) in shrimp were involved in WSSV infection. Multiple envelope proteins of WSSV containing the RGD motif are involved in WSSV infection [12]. Moreover, it has been demonstrated that a β-integrin of *Marsupenaeus japonicus* could block the WSSV infection in vivo and in vitro by binding to VP187, which contains an RGD motif [13]. Meanwhile, our previous study also showed that the extracellular region of β-integrin of Chinese shrimp (*Fenneropenaeus chinensis*) (FcβInt-ER) could specifically bind to WSSV, and in vivo neutralization assay also showed that FcβInt-ER could block WSSV infection [14]. Taken together, these results suggest that the interaction of integrins with WSSV or its RGD-contained envelope proteins plays important roles in WSSV infection.

In order to further clarify the interaction between FcβInt and WSSV and the potential roles of FcβInt in WSSV infection, the binding specificity of rFcβInt to WSSV envelope proteins was investigated by far-western, and the blocking effects of the anti-rFcβInt antibody were also detected both in vitro and in vivo. Meanwhile, the eukaryotic expression plasmid PcDNA3.1-FcβInt-ER (PFcβInt-ER) was constructed to investigate the potential role of PFcβInt-ER in WSSV infection. Furthermore, gene silencing technology was also employed to verify the effect of FcβInt in WSSV infection.

## 2. Results

### 2.1. Interaction between rFcβInt-ER and Five WSSV Envelope Proteins

**Figure 1:**
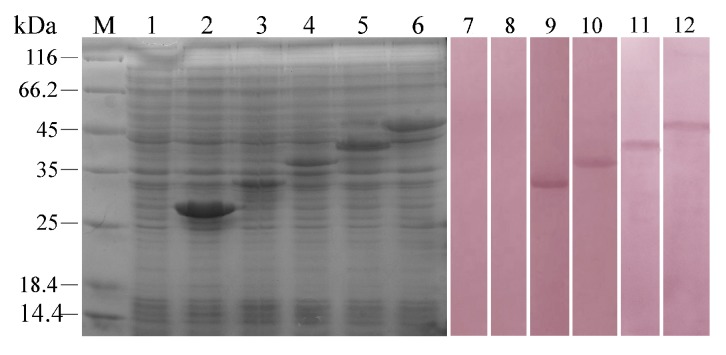
Detection of the binding specificity of rFcβInt-ER to five WSSV envelope proteins by far-western assay. Lane M, molecular weight protein marker; Lane 1–6: The uninduced bacteria lysate and five recombinant envelope proteins stained by Coomassie Brilliant blue. Lane 7–12: Western blotting of five recombinant envelope proteins on PVDF membranes. Lane 1 and 7: Negative controls using the uninduced bacteria lysate; lane 2 and 8: rVP28; lane 3 and 9: rVP31; lane 4 and 10: rVP37; lane 5 and 11: rVP110; lane 6 and 12: rVP187.

To identify whether rFcβInt-ER could bind to the RGD-containing envelope proteins of WSSV, recombinant VP28, VP31, VP37, VP110 and VP187 were produced in an *E. coli* expression system with the predicted molecular masses of 27, 34, 36, 42 and 47 kDa, respectively. After IPTG induction, whole cell lysates were analyzed by SDS-PAGE, and bands matching the expected molecular weights were observed by Coomassie Blue staining (Figure 1). In order to determine the interactions between rFcβInt-ER and the recombinant WSSV envelope proteins, the purified rFcβInt was used as a probe to react with the recombinant envelope proteins by far-western. It was shown that rFcβInt-ER was able to specifically interact with rVP31, rVP37, rVP110 and rVP187 except rVP28 (Figure 1). No positive reaction was observed in the negative control.

### 2.2. Blocking Effects of Anti-rFcβInt-ER Antibodies In Vitro

**Figure 2:**
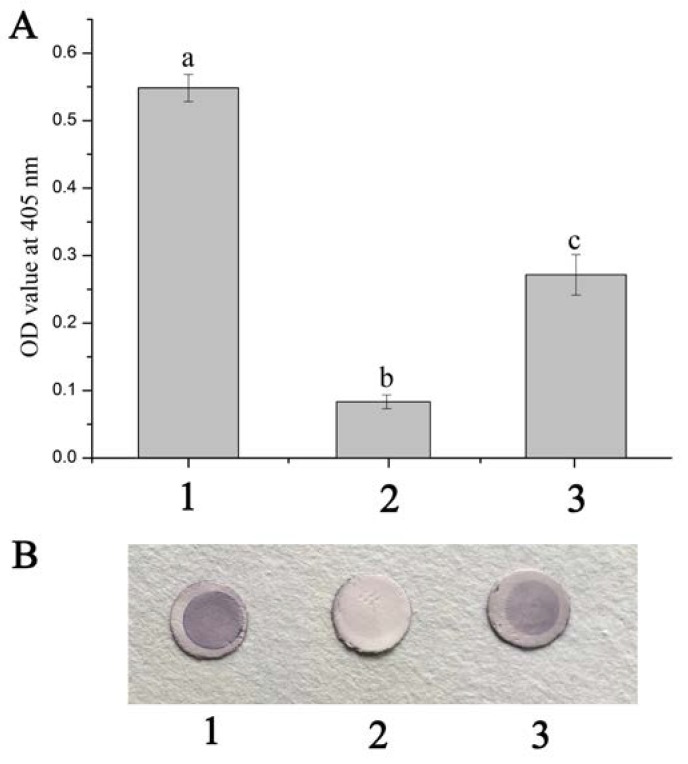
Detection of the blocking effects of anti-rFcβInt-ER antibodies by ELISA (**A**) and dot-blot (**B**). 1: HmFc incubated with WSSV-DIG as positive control; 2: HmFc pre-incubated with anti-rFcβInt-ER antibodies was then incubated with WSSV-DIG; 3: PBS instead of WSSV-DIG as negative control. Different letters on the bar represent the statistical significance (*p* < 0.05) compared to the other groups.

The ELISA reading of the positive control exceeded the reading of negative control (P/N > 2.1), which revealed that WSSV could bind to the hemocyte membrane of *F. chinensis* (HmFc). While the HmFc were pre-incubated with anti-rFcβInt-ER antibodies and then incubated with WSSV-DIG, the OD value was significantly reduced, which indicated that the binding of WSSV to HmFc was partially blocked by the anti-rFcβInt-ER antibodies (Figure 2A). In the dot-blotting assay, the blocking effect of anti-rFcβInt-ER antibodies was also detected. Compared with the positive control, a lighter color was observed when WSSV-DIG was incubated with the HmFc pre-incubated with anti-rFcβInt-ER antibodies, and there was no color present in the negative control (Figure 2B).

### 2.3. Infection-Blocking Effect of Anti-rFcβInt-ER Antibodies In Vivo

**Figure 3:**
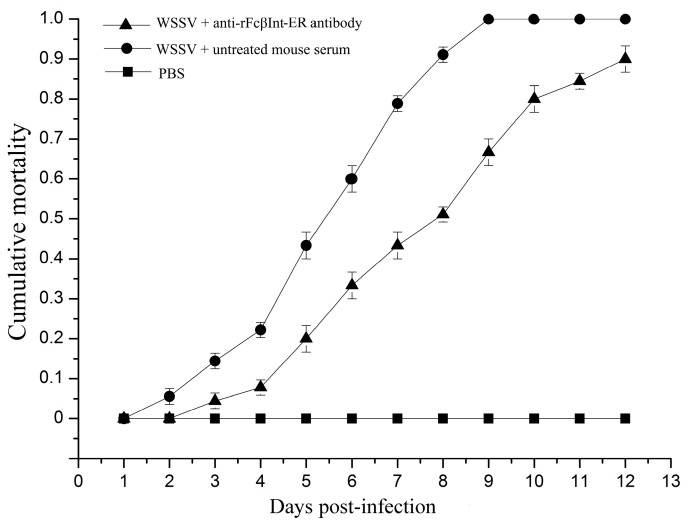
In vivo WSSV infection blocking assay with anti-rFcβInt-ER antibodies. Shrimp in all groups were challenged with WSSV, and the mortalities were recorded for 12 days. Shrimp injected with PBS were set as control. The bar represents the SD of the mean (*n* = 3).

To investigate whether FcβInt play roles in WSSV infection, an in vivo blocking assay using anti-rFcβInt-ER antibodies was performed in *F. chinensis*. After WSSV challenge, the mortality of shrimp injected with WSSV mixed with untreated mouse serum increased steadily from the 2nd day post-infection, and reached 100% at the 9th day in the positive control group. By contrast, no mortality was observed in the control shrimp injected with PBS. When the shrimp were challenged with WSSV mixed with anti-rFcβInt antibodies, the mortality was delayed, and lower than in the control group, which finally reached 90% at the 12th day post-infection (Figure 3). All the dead shrimp were WSSV-positive by PCR (data not shown). These results indicated that anti-rFcβInt-ER antibodies could partially block WSSV infection.

### 2.4. Tissue Distribution and In Vivo Transcription Analysis of PFcβInt-ER in F. chinensis

**Figure 4:**
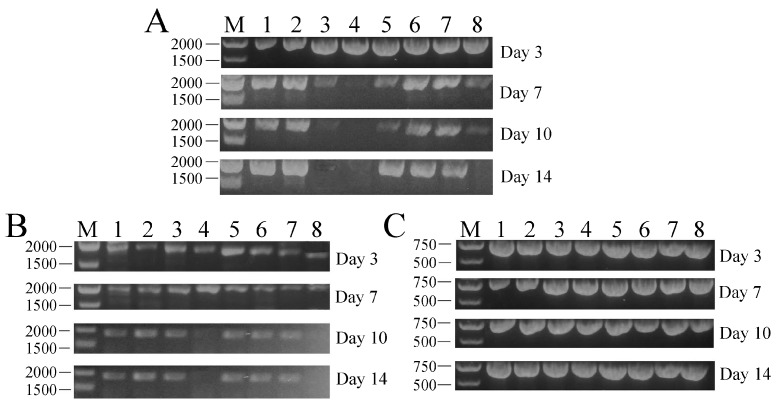
Analysis of the tissue distribution (**A**), and in vivo transcription (**B**,**C**), in *F. chinensis* injected with PFcβInt-ER. (**A**) tissue distribution of PFcβInt-ER in shrimp injected with the expression vector at different times by PCR analysis; (**B**) in vivo transcription of PFcβInt-ER in shrimp injected with the expression vector at different times by RT-PCR analysis; (**C**) in vivo transcription of β-actin in shrimp injected with the expression vector at different times by RT-PCR analysis. (Lane M: DNA marker; 1: heart; 2: gill; 3: stomach; 4: hemolymph; 5: intestine; 6: muscle; 7: gonad; 8: hepatopancreas.)

PCR was employed to investigate the presence of the plasmid PFcβInt-ER at remote sites of injection. Various tissues including heart, gill, stomach, hemolymph, intestine, muscle, gonad and hepatopancreas were collected for detection of plasmid DNA. The results showed that PFcβInt-ER could be detected in all the tissue samples examined at day 7 after injection. On day 10 after injection, PFcβInt-ER could not be detected in hemolymph, and its content was also very low in the stomach, intestine and hepatopancreas. On day 14 after injection, PFcβInt-ER could not be detected in hemolymph and hepatopancreas (Figure 4A). No positive band was detected in the tissues of control shrimp injected with PBS (data not shown).

The transcription of PFcβInt-ER was also detected by RT-PCR analysis in total RNA extracted from different tissues on days 3, 7, 10 and 14, post-injection, with PFcβInt-ER. The results showed that the transcription levels were different among the different tissues; the transcripts of PFcβInt-ER could be detected in all the examined tissues on days 3 and 7, whereas which were absent in hepatopancreas and hemolymph since the 10th day post-injection (Figure 4B). Meanwhile, the β-actin transcripts maintained stable levels in all the detected tissues throughout the whole experimental period (Figure 4C).

### 2.5. Neutralizing Effect of PFcβInt-ER against WSSV Infection

**Figure 5:**
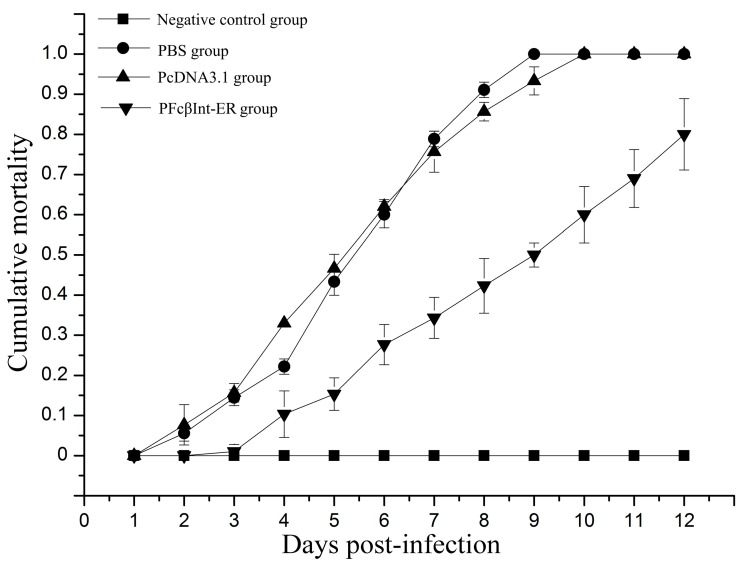
In vivo neutralization assay of WSSV infection with PFcβInt-ER. All groups challenged with WSSV 10^5^ virions per shrimp and their mortalities were recorded for 12 days. Shrimp were treated with PBS as a control. The bar represents the SD of the mean (*n* = 3).

The groups of shrimp injected intramuscularly with PFcβInt-ER, PcDNA3.1 or PBS were challenged with WSSV on the 7th day post-injection. On the 9th day post-challenge, the survival rate of the shrimp injected with PFcβInt-ER reached 50.0%, whereas 100% mortality was observed in the PBS control group, and 93.3% mortality was observed in the PcDNA3.1 control group (Figure 5). On the 12th day post-challenge, the mortality of shrimp injected with PFcβInt-ER reached 80%, whereas 100% mortality was observed in both the PBS control group and the PcDNA3.1 control group. By contrast, no mortality of shrimp challenged with PBS was observed, and all the dead shrimp were WSSV-positive by PCR (data not shown). These results demonstrated that injection with the PFcβInt-ER vector could partially protect shrimp from WSSV infection.

### 2.6. Gene Silence of FcβInt by dsRNA-Mediated RNAi

**Figure 6:**
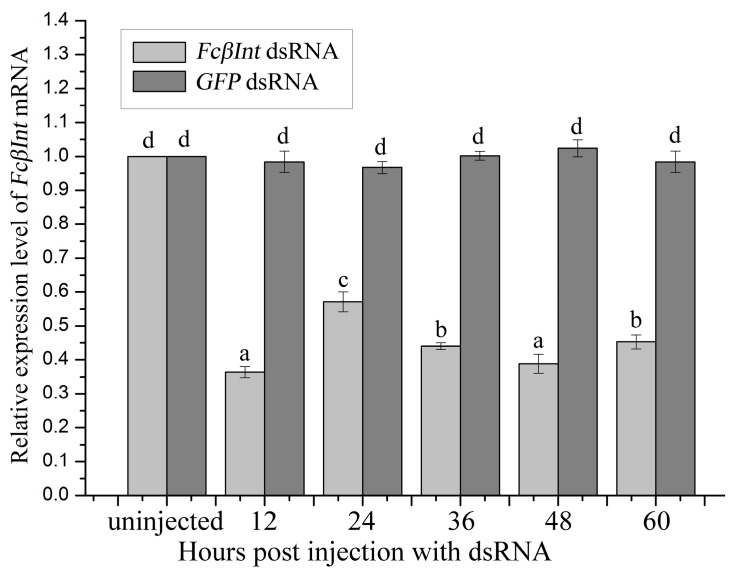
Effects of dsRNA-mediated FcβInt silencing in shrimp hemocytes detected by qRT-PCR. Significant differences (*p* < 0.05) between groups are marked with different letters above bars. Each symbol and vertical bars represented the mean ± SD (*n* = 3).

The RNAi experiment was performed with the shrimp *F. chinensis* injected with dsRNA for FcβInt or GFP. For the shrimp that received FcβInt dsRNA, the FcβInt mRNA level in hemocytes was silenced by 64% at 12 h post-injection; however, it bounced back to about 43% at 24 h post-injection compared to the control group. When a second injection with FcβInt dsRNA was administered, FcβInt mRNA expression was silenced and maintained at a lower level at approximately 44%, 39% and 45% of the control level at 36, 48 and 60 h post-injection, respectively (Figure 6). For the shrimp that received GFP dsRNA, the FcβInt mRNA in hemocytes did not show significant change at any detection time.

### 2.7. Inhibition of WSSV Infection after Silencing the FcβInt Gene

**Figure 7:**
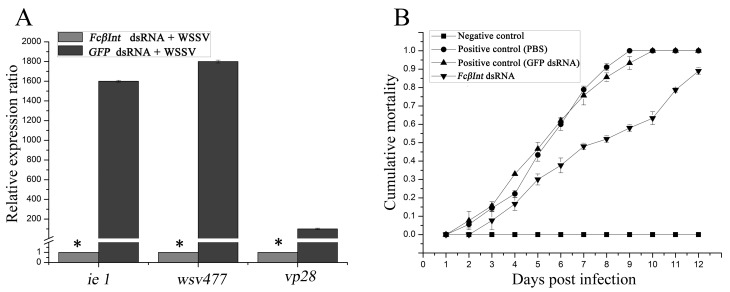
Influence of dsRNA-mediated FcβInt silence on WSSV genes expression in hemocytes (**A**) and mortalities of viral-challenged shrimp (**B**). Significant differences (*p* < 0.05) are marked with an asterisk. The bar represents the SD of the mean (*n* = 3).

When shrimp were challenged with WSSV at 12 h after the second injection of dsRNA, the expression levels of the WSSV immediate early gene *iel*, early gene *wsv477*, and late gene *VP28* all significantly decreased by more than 95% in the FcβInt knock-down hemocyctes compared with the control shrimp (Figure 7A). These observations showed that RNAi-mediated FcβInt transcript suppression reduced WSSV gene transcripts in the hemocytes of WSSV-infected *F. chinensis*. Moreover, the survival rate of shrimp pretreated with integrin-specific dsRNA reached 42%, whereas 100% mortality was observed in the PBS control group, and 93.3% mortality was observed in the GFP dsRNA control group on the 9th day post-challenge (Figure 7B). On the 12th day post-challenge, the mortality of shrimp injected with integrin-specific dsRNA reached 87%, whereas 100% mortality was observed in both the PBS control group and the GFP dsRNA control group. No shrimp died in the negative control injected with PBS instead of WSSV, and all the dead shrimp were WSSV-positive by PCR (data not shown).

## 3. Discussion

Integrins could recognize multiple ligands and mediate cell-cell and cell-extracellular matrix interactions [3]. The RGD motif is the cell attachment site of a large number of adhesive extracellular matrix, and cell surface proteins, and nearly half of the over 20 known integrins recognize this motif in their adhesion protein ligands [15]. Likewise, the conserved RGD tripeptide was shown to play important roles in virus infectivity which could be binding to integrins [16]. A large number of viruses and bacteria infect their host by binding to integrins with canonical integrin-binding motifs [17]. Previous studies have shown that many envelope proteins of WSSV contain the RGD motif, and recent studies have further shown that integrins in shrimp were involved in WSSV infection [11,18]. Our previous study has shown that an integrin β-subunit of *F. chinensis* could specifically bind to the WSSV and involved in WSSV infection [14]. However, the research of the interaction between the β-integrin of *F. chinensis* and WSSV envelope proteins is still limited. In this study, the results of far-western showed that rFcβInt-ER was able to recognize the rVP31, rVP37, rVP110 and rVP187, which all contain cell attachment RGD motif. Among them, VP37 is an important envelope protein of WSSV [19], which plays a major role in WSSV infection [20], and the recombinant VP37 has shown a high binding activity with the shrimp cell membrane in a binding assay [21]. VP31 is also an important viral envelope protein that might be involved in WSSV infection [22], and was identified as being able to interact with several proteins in shrimp [23,24]. VP110 was able to bind to the surface of crayfish haemocyte, and the synthetic RGDT peptide was able to inhibit attachment of VP110 to the cell membrane [25]. These results indicated that the WSSV envelope proteins possessing the RGD motif play important roles in WSSV infection. Therefore, we suspect that the β-intergrin of *F. chinensis* serves as a critical receptor for WSSV attachment and entry. It’s also worth noting that VP28 that does not contain RGD motif plays an important role in WSSV infection [26], which indicates that there might be other infection mechanisms employed by WSSV.

It is well known that receptor proteins play an important role in the interaction of viruses and cells. Integrin, an important cell membrane receptor, plays important roles in many virus infections [5,27,28]. We have previously demonstrated that the β-integrin of *F. chinensis* (FcβInt) can specifically bind to WSSV, and the extracellular region of FcβInt (FcβInt-ER) could partial block the infection of WSSV in vivo [14]. In the present study, infection-blocking assays showed that anti-FcβInt-ER antibodies could block WSSV binding to HmFc in vitro in ELISA and dot-blot assays, and partially block WSSV infection of *F. chinensis* in vivo in the neutralization assay. Meanwhile, the blocking effect of FcβInt-ER was also determined in vivo by injecting the eukaryotic expression plasmid PcDNA3.1-FcβInt-ER. Consistent with our findings, previous studies have also shown that the WSSV infection could be partially blocked by the recombinant integrin and its antibodies in vitro and in vivo in *M. japonicus* [13]. Furthermore, WSSV pre-incubated with *L. vannamei* β-integrin before injecting showed lower mortality rate in vivo [9]. All of these results indicated that integrins play important roles in WSSV infection, and might serve as receptors or “post-attachment” receptors or co-receptors for WSSV attachment and entry. It is worth noting that the blocking is incomplete, suggesting that integrin is not the only receptor for WSSV.

Integrins, as important cell adhesion receptors, not only play an important role in mediating cell adhesion, but also play an important role in cell bidirectional signal transduction [29]. The activation of integrin signaling pathways could regulate multiple cell functions by triggering a large variety of signal transduction events, including proliferation, survival/apoptosis, shape, polarity, motility, gene expression and differentiation [4]. The process of coelomocyte apoptosis was significantly promoted by injecting a β-integrin specific siRNA in *Apostichopus japonicus* in vitro [30]. Likewise, it has been shown that the decreased expression of integrin-β1 by injection of double stranded integrin-β1 RNA could significantly suppress the capacity of plasmatocyte encapsulation in *Manduca sexta* [31]. Recently, studies have also shown that the infection of WSSV could activate the integrin signaling pathway by phosphorylating its downstream signal molecules, and the activation of the integrin signaling pathway could inhibit *M. japonicus* hemocyte apoptosis for viral propagation [32]. In the present study, we also found that the infection of WSSV was significantly inhibited by knocking down the expression of FcβInt using RNA interference technology. Similarly, integrin gene silencing mediated by an integrin-specific dsRNA effectively inhibited WSSV infection in *M. Japonicus* [13]. Moreover, our previous study showed that the expression of β-integrin in *F. chinensis* was significantly upregulated after WSSV challenge [14]. These results indicated that the up-regulated expression of β-integrin post WSSV infection would promote the activation of integrin signaling pathway that facilitated WSSV proliferation.

## 4. Materials and Methods

### 4.1. Expression of Five WSSV Envelope Proteins

The virus was extracted from the gills of naturally WSSV-infected *F. chinensis* according to previous methods [33]. The virus pellets were suspended in a TNE buffer (50 mM Tris, 100 mM NaCl, 10 mM EDTA, pH 7.4) and analyzed by transmission electron microscope (TEM) to test the integrity. WSSV-DNA was extracted from the viral suspensions following the method described previously [34]. The WSSV genes *vp28*, *vp31*, *vp37*, *vp110*, *vp187* were cloned individually onto the BamH I and Xho I sites of the T7 expression vector pET-28a (+). Each gene was amplified by PCR from WSSV-DNA with primers carrying restriction sites for BamH I and Xho I (Table 1), and restricted with BamH I and Xho I restriction endonucleases and ligated into pET-28a (+) digested with the same enzymes, using T4 DNA ligase. The recombinant plasmids were then expressed in *E. coli* BL21 (DE3) and confirmed by SDS-PAGE.

### 4.2. Detection of Binding Specificity of rFcβInt-ER to WSSV Envelope Proteins by Far-Western

The extracellular region of the β-integrin of *F. chinensis* (FcβInt-ER) was expressed, and anti-FcβInt-ER polyclonal antibodies were produced in Kunming mice, immunized as previously described [14]. To confirm the interaction of rFcβInt-ER with the envelope proteins of WSSV, a far-western assay was performed. Briefly, whole cells lysates of induced *E. coli* expressing WSSV envelope Protein VP28, VP31, VP37, VP110 and VP187 were subjected to SDS-PAGE analysis, and then the separated proteins were transferred onto PVDF membranes and blocked with phosphate buffered saline tween (PBST) containing 3% bovine serum albumin (BSA). Then, the blocked membranes were incubated with rFcβInt-ER for 2 h at 37 °C. After washing thrice, the membranes were incubated for 1 h at 37 °C with monoclonal antibody (mAb) 2C5, which was produced previously, and was able to specifically react with the β-integrin of *F. chinensis* [35]. Then, the membrane was incubated with alkaline phosphatase (AP)-conjugated goat anti-mouse Ig antibody (1:4000, Sigma-Aldrich, St. Louis, MO, USA) at 37 °C for 1 h. Finally, immunoreactions were visualized by NBT-BCIP (Sigma, St. Louis, MO, USA) staining and stopped with distill water. In negative control, the rFcβInt-ER was replaced by His-tag.

### 4.3. Preparation of HmFc and Digoxigenin (DIG) Labeled WSSV (WSSV-DIG)

The hemocyte membrane of *F. chinensis* (HmFc) was prepared as described previously [36]. Briefly, healthy Chinese shrimp were collected from the Yellow Sea and detected to be WSSV-free, and were then maintained at 25 ± 1 °C in tanks. After one-week acclimatization, the hemolymphs were withdrawn from the pericardial cavity with 1:1 precooled (4 °C) anticoagulant (9 mM EDTA, 336 mM NaCl, 115 mM glucose, 27 mM Nacitrate, pH 7.0). After centrifugation at 600× *g* at 4 °C for 10 min, the haemocytes were collected and homogenized in precooled lyses buffer (10% sucrose (*w*/*v*) containing, 2 mM PMSF, 2 mM EDTA, 10 mM Hepes, pH 7.4). Then, the homogenate was centrifuged at 8000× *g* at 4 °C for 10 min to get rid of the unbroken cells and cellular organelles. Finally, the pellets of hemocyte membrane was obtained from supernatant fluid by ultracentrifuging at 100,000× *g* at 4 °C for 20 min (Hitachi CP 100MX, Tokyo, Japan). The pellets were re-suspended with PBS (1.47 mM KH_2_PO_4_, 8.09 mM, 2.7 mM KCl, Na_2_HPO_4_, 137 mM NaCl, pH 7.4) and adjusted to 1 mg mL^−1^ with PBS containing 1% bovine serum albumin (BSA). WSSV virions were labeled with DIG using the DIG-Protein Labeling Kit according to the manufacturer’s instructions (Roche, Berlin, Germany).

### 4.4. Blocking Effects of Anti-rFcβInt-ER Antibodies In Vitro and In Vivo

Blocking assays in vitro were carried out by ELISA and dot-blotting. For ELISA, HmFc suspensions were dropped into 96-Well microtiter plates and incubated at 37 °C for 2 h, the wells were washed thrice with PBST and then blocked with 5% BSA in PBS buffer for 1 h at 37 °C and subsequently incubated with mouse anti-rFcβInt-ER sera (1:100 dilution in PBS) for 2 h at 37 °C. The wells were washed as above and incubated with WSSV-DIG for 2 h at room temperature. After washing as above, the wells were incubated with an AP-conjugated anti-DIG antibody (Roche, Germany) for 1 h at 37 °C, washed again and developed with *p*-nitrophenylphosphate. The reaction was stopped by adding 2 M NaOH and its absorbance was read at 405 nm. Untreated mouse serum instead of mouse anti-FcβInt-ER sera were performed as negative control, and PBS instead of WSSV-DIG was set as another negative control. Experiments were performed in triplicate for statistical analysis.

For dot-blotting, 4 μL of HmFc suspensions (100 μg mL^−1^) was spotted onto 6-mm-diameter nitrocellulose membranes located in the wells of 96-well microplate and air-dried, then blocked overnight at 4 °C with 3% BSA in PBS. After washing thrice with PBST, 100 μL anti-rFcβInt-ER sera (1:100 diluton in PBS) was added and incubated for 2 h at 37 °C. After washing thrice, 100 μL WSSV-DIG was added and incubated for 2 h at 37 °C. The membranes were washed as above, and incubated with an AP-conjugated anti-DIG antibody (Roche, Germany) for 1 h at 37 °C. After washing as above, the alkaline phosphatase reaction was developed in a substrate solution of NBT-BCIP (Sigma, St. Louis, MO, USA) for about 15 min. The untreated mouse serum instead of anti-FcβInt-ER antibodies were used as negative control, and PBS instead of WSSV-DIG was set as another negative control.

In order to investigate whether anti-FcβInt-ER antibodies could block the infection of WSSV in vivo, an infection-blocking assay was performed on shrimps. Adult Chinese shrimp with a length of 15 ± 2 cm were captured from the Yellow Sea and detected to be WSSV-free, and then maintained in aerated seawater at 25 ± 1 °C for a week before use. The injecting dose was determined by injecting shrimp with serial dilutions of virions, the results showed that shrimp will be infected and die in 7–10 days with an optimal injection dose (1 × 10^5^ virions per shrimp) (data not shown). Prior to injection, WSSV virions were mixed with mouse anti-FcβInt-ER sera (1:100 diluton in PBS). WSSV mixed with untreated mouse serum was used as a positive control. Shrimps injected with PBS was performed as a negative control. Then, a dose of 100 μL of the various mixtures was intramuscularly injected into each shrimp of the three groups. Each treatment contained 30 shrimp, with three replicates. Mortality was recorded daily, and WSSV was detected in the gills of dead shrimp by PCR as previous described [34].

### 4.5. Preparation of Eukaryotic Expression Plasmid PcDNA3.1-FcβInt-ER (PFcβInt-ER)

The extracellular region of the β-integrin of *F. chinensis* with signal peptide was amplified by PCR with specific primers: the forward primer (5′-CGCGGTACCACCATGAAGGCGGGCATCCCCTTGG-3′) including an Kpn I restriction site and a Kozak sequence (CCACC), and the reverse primer (5′-TTGCGGCCGCTCAAGGTGCAGCTTCCGGACACTG-3′) containing a Not I site. The PCR product of FcβInt-ER was cloned into the PMD-19T vector (Baosheng, Dalian, China) and sequenced, and then ligated to the Kpn I/Not I-digested pcDNA3.1 expression vector (Invitrogen, Carlsbad, CA, USA). The recombinant expression plasmid was named PFcβInt-ER, and transformed to *E. coli* DH5α for propagation. Then, the recombinant plasmids were examined by restriction digestion and sequencing. For the purpose of vaccination, both the empty expression vector pcDNA3.1 and the recombinant plasmids PFcβInt-ER were extracted using the plasmid purification kit (Qiagen, Hilden, Germany) following the manufacturer’s instructions. The extracted plasmids were aliquoted at 600 ng μL^−1^ in sterile endotoxin-free PBS, and stored at −20 °C until further use.

### 4.6. PCR Detection of Plasmid DNA and In Vivo Transcriptional Analysis of PFcβInt-ER in F. chinensis

For PFcβInt-ER distribution and transcriptional analyses, two groups of shrimp were injected intramuscularly with plasmid DNA (PFcβInt-ER) (30 μg per shrimp) and tissue samples (heart, gill, hepatopancreas, stomach, intestines, gonad and muscle) were collected at Day 3, 7, 10 and 14 post-injection. For tissue distribution analysis, the whole DNA of various tissues was isolated with DNeasy Blood & Tissue Kit (Qiagen, Hilden, Germany) as per the manufacturer’s instructions. The extracted DNA was dissolved in nuclease-free water and subjected to PCR analysis to determine the distribution of PFcβInt-ER with a universal primer set of PcDNA3.1 plasmid designated as T7-F and BGH-R in Table 1, and then the PCR products were separated on 2.0% agarose gels. In order to detect the transcription of PFcβInt-ER, the total RNA was extracted from different tissues using Trizol reagent (Invitrogen Life Technologies, Carlsbad, CA, USA) and treated with DNase I (Baosheng, Dalian, China) for 30 min at 37 °C to remove the genomic DNA according to the manufacturer’s instruction. Then, total RNA was quantified by a Nanodrop 8000™ Spectrophotometer (Thermo Scientific, Waltham, MA, USA). Single-strand cDNA was synthesized from 1 µg total RNA using PrimeScript™ RT-PCR Kit (Baosheng, Dalian, China) according to the manufacturer’s instructions. The cDNA reaction products were subjected to PCR with a universal primer set of PcDNA3.1 plasmid as above. The shrimp β-actin gene (F: 5′-AGTCTAACGCGGGTACTCCCTCTGT-3′; R: 5′-CTCCTTGATGTCACGCACGATTTCT-3′) was used as an internal control for RT-PCR.

### 4.7. Neutralization Assay In Vivo Using PFcβInt-ER

Shrimp were divided into three groups, which were kept separately for experiments. The first group of shrimp was injected intramuscularly by a single dose of 30 μg plasmid DNA. The second group of shrimp was injected with the same volume of PBS and the third group of shrimp was injected with 30 μg of pcDNA3.1. On the 7th day post-injection, shrimp were challenged with WSSV following the procedure described above. Mortality was recorded daily to determine the neutralization effect of PFcβInt-ER.

### 4.8. Gene Silencing of FcβInt

Based on the protocols described by Lin et al. [37] and the online software (http://www.dkfz.de/signaling/e-rnai3//), the open reading frame (ORF) of FcβInt dsRNA from 1650 to 2320 bp was designed using T7 RiboMAX (Promega, Madison, WI, USA). Agarose gel electrophoresis was performed to examine the synthesized dsRNA. Then, the concentration of dsRNA was quantified by Nanodrop 8000™ (Thermo Scientific, Waltham, MA, USA). The synthesized GFP dsRNA was used as a control. The primers used to synthesize GFP dsRNA and FcβInt dsRNA are all listed in Table 1.

To estimate the FcβInt silencing effect, shrimp were injected intramuscularly with FcβInt dsRNA at a dose of 1 μg/g per shrimp, and the shrimps injected with equivalent amounts of GFP dsRNA were set as control. At 24 h after the first injection, a booster injection was administrated as described by Feng et al. [38]. At 12, 24, 36, 48 and 60 h after the first injection, 3 shrimp were randomly selected from the groups of FcβInt and GFP dsRNA for hemocytes isolation. The silencing effect on FcβInt was examined at mRNA level by qRT-PCR, and the β-actin gene was used as internal control.

In order to investigate the effect of FcβInt transcript suppression on WSSV infection, the transcription levels of three WSSV genes were determined in the FcβInt silenced shrimps after WSSV infection. Based on the silencing effect on FcβInt after injection of dsRNA, all the experimental shrimp were challenged with WSSV at 12 h after the second injection of dsRNA, according to the procedure described above. The total RNA was extracted from the collected hemocytes at 24 h after WSSV infection, and the expression levels of WSSV immediate early gene *ie1*, early gene *wsv477*, and late gene *VP28* were examined in both the FcβInt silenced and control shrimps by qRT-PCR. The primers used were designed according to the previous work by Yingvilasprasert et al. [39]. The β-actin gene was used as an internal control. The primers used in this part were listed in Table 1. Meanwhile, the effects of gene silencing of FcβInt dsRNA on WSSV infection in vivo were investigated. Three groups were injected with FcβInt dsRNA, GFP dsRNA and PBS, respectively. Shrimp were challenged with WSSV following the procedure described above at 12 h post the second injection of dsRNA. Nothing was injected to the fourth group, as an absolute control. Mortality was recorded daily for 12 days.

### 4.9. Statistical Analysis

The relative gene expression values were analyzed using the 2^−ΔΔ*C*t^ method. All the gene expression levels were determined in three replicates of qRT-PCR experiment. The statistical analysis was performed using Statistical Product and Service Solution (SPSS) software(Version 17.0; SPSS Inc., IBM, Armonk, NY, USA), differences were analyzed with one-way analysis of variance (ANOVA) and the results were expressed as means ± standard deviation (SD) (*n* = 3). In all cases, the significance level was defined as *p* < 0.05.

## 5. Conclusions

In conclusion, the β-integrin of *F. chinensis* were able to interact with four envelope proteins of WSSV containing the RGD motif, including rVP31, rVP37, rVP110 and rVP187. The antibodies against rFcβInt-ER were able to partially block the binding of WSSV to the hemocyte membrane in vitro, and inhibit WSSV infection in vivo. The eukaryotic expression plasmid PFcβInt-ER has a partial neutralization effect on WSSV infection in shrimp. Furthermore, the mortality of *F. Chinensis* was also significantly decreased in the FcβInt silenced shrimp than the control group after WSSV challenge. Taken together, these results suggest that the β-integrin serves as a receptor for WSSV infection.

## Figures and Tables

**Table 1:** Primers used in this study.

Sequence Information	Primers Name	Sequence of Primers (5′-3′)
CDs of WSSV envelope Protein vp28	VP28-F	CGGGATCCATGGATCTTTCTTTCACTC
	VP28-R	GGCCTCGAGCTCGGTCTCAGTGCCAG
CDs of WSSV envelope Protein vp31	VP31-F	CGGGATCCATGTCTAATGGCGCAAC
	VP31-R	GGCCTCGAGCTCCTCCTTAAAAGCAGT
CDs of WSSV envelope Protein vp37	VP37-F	CGGGATCCATGGCGGTAAACTTGG
	VP37-R	GGCCTCGAGTGTCCAACAATTTAAAAAG
Partial sequence of WSSV envelope Protein vp110 including RGD motif	VP110-F	CGGGATCCACCCACAAAGGACCACC
	VP110-R	GGCCTCGAGGTCCCTTATTTCTTCCAG
Partial sequence of WSSV envelope Protein vp187 including RGD motif	VP187-F	CGGGATCCGACGACGTTACAAATTTAC
	VP187-R	GGCCTCGAGCTGAGAGAGGGCACCCGAGC
Universal primer set of PcDNA3.1 plasmid	T7-F	TAATACGACTCACTATAGGG
	BGH-R	TAGAAGGCACAGTCGAGG
Gene silencing of FcβInt (FcβInt dsRNA)	FcβIntRNAiT7-F	taatacgactcactatagggGTGTTCTGGTCACGGGACTT
	FcβIntRNAi-R	TGAATGTGTTGGTTGCAGGT
	FcβIntRNAi-F	GTGTTCTGGTCACGGGACTT
	FcβIntRNAiT7-R	taatacgactcactatagggTGAATGTGTTGGTTGCAGGT
Gene silencing control (GFP dsRNA)	eGFPRNAiT7-F	taatacgactcactatagggGACGTAAACGGCCACAAGTT
	eGFPRNAi-R	TGTTCTGCTGGTAGTGGTCG
	eGFPRNAi-F	GACGTAAACGGCCACAAGTT
	eGFPRNAiT7-R	taatacgactcactatagggTGTTCTGCTGGTAGTGGTCG
QPCR for *FcβInt*	FcβInt-QF	GACCCGCTGAGTGATGTTTC
	FcβInt-QR	CTTGAACTGCGTCGTGAGG
QPCR for *β-actin*	β-actin-QF	GAAGTAGCCGCCCTGGTTG
	β-actin-QR	GGATACCTCGCTTGCTCTGG
QPCR for *ie1*	ie1-QF	AGCAAGTGGAGGTGCTATGT
	ie1-QR	CCATGTCGATCAGTCTCTTC
QPCR for *wsv477*	wsv477-QF	GGCCAAGTCATGGAGATCTA
	wsv477-QR	CCATCCACTTGGTTGCAGTA
QPCR for *vp28*	vp28-QF	GGGAACATTCAAGGTGTGGA
	vp28-QR	GGTGAAGGAGGAGGTGTTGG
